# Toward Detection of the Molecular Parity Violation in Chiral Ru(acac)_3_ and Os(acac)_3_

**DOI:** 10.1021/acs.jpclett.2c02434

**Published:** 2022-10-20

**Authors:** Marit R. Fiechter, Pi A. B. Haase, Nidal Saleh, Pascale Soulard, Benoît Tremblay, Remco W. A. Havenith, Rob G. E. Timmermans, Peter Schwerdtfeger, Jeanne Crassous, Benoît Darquié, Lukáš F. Pašteka, Anastasia Borschevsky

**Affiliations:** † Van Swinderen Institute for Particle Physics and Gravity (VSI), University of Groningen, Nijenborgh 4, 9747 AG Groningen, The Netherlands; ‡ Department of Physics, ETH Zürich, Otto-Stern-Weg 1, 8093 Zurich, Switzerland; ¶ Department of Organic Chemistry, University of Geneva, Quai Ernest Ansermet 30, 1211 Geneva 4, Switzerland; § Université de Rennes, CNRS, ISCR-UMR 6226, Campus de Beaulieu, 35042 Rennes Cedex, France; ∥ Sorbonne Université, CNRS, UMR 8233, MONARIS, Case courrier 49, 4 place Jussieu, F-75005 Paris, France; ⊥ Zernike Institute for Advanced Materials, University of Groningen, Nijenborgh 4, 9747 AG Groningen, The Netherlands; # Stratingh Institute for Chemistry, University of Groningen, Nijenborgh 4, 9747 AG Groningen, The Netherlands; @ Ghent Quantum Chemistry Group, Department of Chemistry, Ghent University, Krijgslaan 281 (S3), B-9000 Ghent, Belgium; △ Centre for Theoretical Chemistry and Physics, The New Zealand Institute for Advanced Study, Massey University, 0745 Auckland, New Zealand; †† Laboratoire de Physique des Lasers, Université Sorbonne Paris Nord, CNRS, 93430 Villetaneuse, France; ‡‡ Department of Physical and Theoretical Chemistry, Faculty of Natural Sciences, Comenius University, Ilkovičova 6, 84215 Bratislava, Slovakia

## Abstract

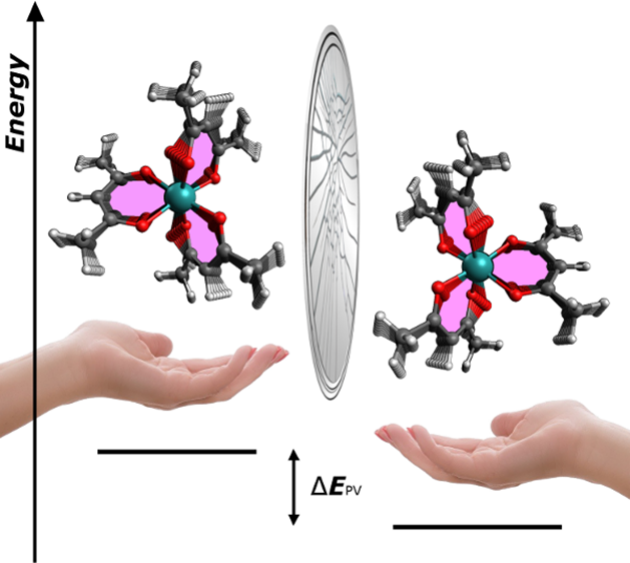

We present a theory-experiment investigation of the helically chiral compounds Ru(acac)_3_ and Os(acac)_3_ as candidates for next-generation experiments for detection of molecular parity violation (PV) in vibrational spectra. We used relativistic density functional theory calculations to identify optimal vibrational modes with expected PV effects exceeding by up to 2 orders of magnitude the projected instrumental sensitivity of the ultrahigh resolution experiment under construction at the Laboratoire de Physique des Lasers in Paris. Preliminary measurements of the vibrational spectrum of Ru(acac)_3_ carried out as the first steps toward the planned experiment are presented.

Parity violation (PV) due to weak interaction was first predicted in 1956,^1^ immediately after being observed in nuclear physics,^2,3^ and later in atomic physics.^4−7^ In chiral molecules, it is predicted to result in a tiny energy difference between enantiomers which, if large enough, may provide the bias needed to seed the observed biomolecular homochirality, i.e. the fact that chiral molecules usually occur in only one enantiomeric form in nature.^8−10^ This PV energy difference can also serve as a sensitive test of the electroweak interactions (to naturally complement low-energy tests using atoms) and of new physics beyond the Standard Model. It is predicted to be particularly sensitive to parity-violating cosmic fields, which are invoked in different models for cold dark matter or in the Lorentz-invariance violating standard model extensions.^11^

Over the past decades, various experiments have been proposed to observe parity violation in chiral molecules, including measurements of PV frequency shifts in NMR spectroscopy,^12−15^ measurements of the time-dependence of optical activity,^16^ and direct measurement of the absolute PV energy shift of the electronic ground state;^17^ see various reviews in this field.^18−20^ However, for none of the aforementioned experimental schemes have tight experimental upper bounds been reported yet;^18^ this has only been accomplished in the measurements of the PV shift of vibrational frequencies, performed at the Laboratoire de Physique des Lasers (LPL) in Paris,^21−25^ using ultraprecise mid-infrared molecular spectroscopy experiments.

Several molecules have been considered as candidates for experiments at LPL. The first experiments were performed on the C–F stretch vibration in CHFClBr lying conveniently in the CO_2_ laser frequency range,^21,22,26,27^ but they led to a nondetection, with an upper limit of Δν^PV^/ν = 2.5 × 10^–13^, with ν the vibrational transition frequency and Δν^PV^ the parity-violating frequency difference between enantiomers. Later, *ab initio* calculations predicted the PV shift for this transition to be 3 to 4 orders of magnitude smaller.^28−31^ Another fluorohalomethane that has been under investigation, CHFClI, is predicted to have a larger PV shift but is not stable enough for measurements.^23,32^ Other candidate molecules include SeOClI,^33^ N≡WHClI,^34^ and N≡UHXY^35^ (X, Y = F, Cl, Br, I). These systems were predicted to possess vibrational transitions with progressively larger PV shifts, as large as several tens of Hz (or Δν^PV^/ν ∼ 10^–13^) in N≡UHFI. So far, the synthesis of this type of compounds has not been reported. More recently, attention has turned to chiral oxorhenium complexes,^36−38^ but bringing these into the gas phase, which is required for high-precision spectroscopy, remains a challenge given their low stability.

In this paper, we investigate a substantially distinct class of highly promising molecules, namely chiral M(acac)_3_ complexes (where M is a metal and acac stands for acetylacetonate; see Figure 1). Specifically, we focus on Ru(acac)_3_ and its heavier homologue Os(acac)_3_. Ruthenium (*Z* = 44) and osmium (*Z* = 76) have reasonably heavy nuclei, so that in accord with the proposed *Z*^5^ scaling law^35,39,40^ we expect these systems to experience a large absolute PV energy shift. Compared to previously proposed species, these molecules exhibit widely different chemical properties—in particular their propeller-like chiral topology (see Figure 1)—and physicochemical properties allowing a number of the above-mentioned limitations to be overcome. In support of M(acac)_3_ molecules, we note that compared to the more exotic species presented above: (i) these are well-known classical archetype systems in organometallic chemistry,^41^ (ii) they feature a relatively high volatility, (iii) with Ru(acac)_3_ being commercially available in its racemic form and being resolvable into pure Δ and Λ enantiomers at gram scales.^42^ In fact, we have recently demonstrated that Ru(acac)_3_ is stable and robust under evaporation by heating up to at least 200 °C and that it can easily be brought into the gas phase.^43^ This allowed us to seed it in a molecular beam^43^ and in a solid matrix of neon at 3 K, allowing preliminary mid-infrared Fourier transform spectroscopic investigations, as reported in the present work. We thus demonstrate an outstanding level of control of a heavy organometallic chiral candidate species for measuring PV. This indicates that this molecule can be fairly easily brought into the gas phase to allow the production of cold and slow gas samples via buffer-gas cooling in a cryogenic chamber, a method that we have recently demonstrated with other organometallic species.^25,44,45^ Buffer-gas-cooled beams, the latest molecular beam technology at the heart of the LPL apparatus,^25^ will provide the low temperature, low speed, and high intensity needed for measuring PV. All in all, these factors make Ru(acac)_3_ a highly attractive candidate for precision gas-phase spectroscopy experiments.

**Figure 1:**
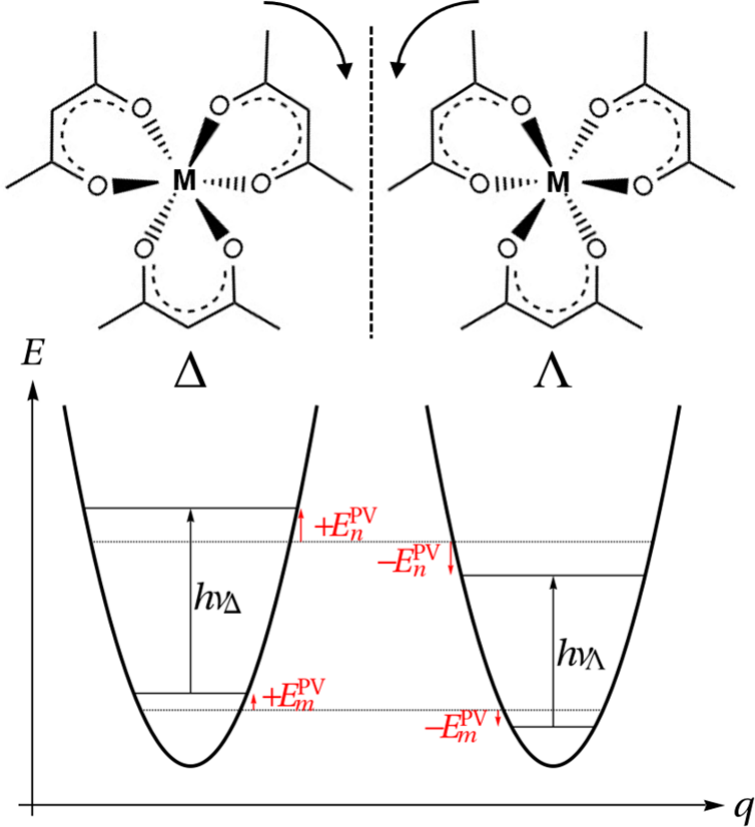
Chemical structures of Δ- and Λ-M(acac)_3_ (M = Ru, Os) with the corresponding transition frequencies ν_Δ_ and ν_Λ_.

The heavier homologue, Os(acac)_3_, is not commercially available but can be synthesized.^46^ Its resolution into pure enantiomers is to be investigated in future experiments. Its higher atomic number will lead to larger vibrational PV shifts,^35^ making it a promising alternative to Ru(acac)_3_.

We perform theoretical investigations of the magnitude of the PV shifts in Ru(acac)_3_ and Os(acac)_3_. Precise spectroscopic data tends to be scarce or simply nonexistent for such species. Here, we present the first spectroscopic investigations of Ru(acac)_3_ that in combination with the calculations allow us to select the most appropriate transitions for measuring parity violation. Such *a priori* investigations are also crucial to determine the frequency range for the metrology-grade laser system to be built and tuned at LPL, as the size of the PV vibrational shift can vary by over an order of magnitude, depending on the vibrational mode.^31^

*Outline of the Parity Violation Calculations*. According to the standard model of particle physics, the dominant *P*-odd contribution of the weak force to the molecular Hamiltonian is due to vector-nucleonic–axial-vector-electronic coupling. In the low-energy limit, the following nuclear spin-independent effective Hamiltonian can be derived from the Standard Model Lagrangian (see e.g. refs (19) and (47)):
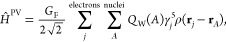
1which is compatible with the usual four-component framework for relativistic quantum chemical calculations. In this equation, *G*_F_ = 2.22255 × 10^–14^ a.u. stands for the Fermi coupling constant; the weak charge of nucleus *A* is given by *Q*_W_(*A*) = [(1–4 sin^2^θ_W_)*Z* – *N*], where θ_W_ is the weak mixing angle and *Z* and *N* are the number of protons and neutrons, respectively; ρ(**r**) stands for the nuclear density; and the fifth gamma matrix can be written in terms of the Dirac matrices as γ^5^ = −*iγ*^0^γ^1^γ^2^γ^3^. The Hamiltonian will yield a contribution to the energy that is positive for one enantiomer and negative for the other.

In this work, we calculate the PV difference in the vibrational transition frequencies between the two enantiomers as illustrated in Figure 1. The computational procedure is structured as follows. The molecular geometries are optimized at the density functional theory (DFT) level, using an effective core potential on the central metal atom to account for scalar relativistic effects. The normal modes and corresponding frequencies are calculated in a harmonic frequency analysis. Subsequently, several normal modes are selected for further fully relativistic calculations; details of the selection criteria are presented later in the text. For each chosen mode, relativistic DFT calculations are performed along the normal mode to obtain the PV potential *V*^PV^(*q*) as a function of the normal coordinate *q*. Full details of the computational setup used in this work (programs, DFT functionals, basis sets, etc.) as well as the investigation of the robustness of this particular computational configuration can be found in the Supporting Information.

Next, the vibrational wave functions |*n*⟩ are determined by solving the vibrational Schrödinger equation numerically following the Numerov–Cooley procedure, for the potential obtained along the normal mode.^48−50^ Then, the shift of a vibrational level *n* can be calculated as
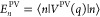
2From this we find the PV frequency difference for a transition from level *m* to level *n* between the left- and right-handed forms of the molecule:
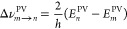
3with *h* being Planck’s constant and where the factor of 2 arises from the fact that the energy of one enantiomer is shifted up by the PV effects, while that of the other is shifted down by the same amount.

In this work, we define the sign of this frequency difference via
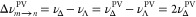
4where ν_Δ_ and ν_Λ_ are the *m* → *n* transition frequencies and ν_Δ_^PV^ and ν_Λ_^PV^ = −ν_Δ_^PV^ stand for the frequency shifts of these transitions in the Δ and Λ enantiomer, respectively (see Figure 1).

As the relativistic calculations are rather expensive computationally, instead of calculating the PV shifts for all normal modes, we select a specific subset of modes for our investigation based on the outcome of the vibrational analysis. In order to have a large differential PV shift, the parity violating energy should vary significantly over the range of a vibration.^35^ The PV effects scale steeply with *Z*, and thus, the metal atom will contribute the most to the total PV energy difference. In order to achieve a large change in the electronic density in the vicinity of the metal atom over the course of a vibration, we look for modes with a large change in the position of the surrounding oxygen atoms. As a measure hereof, we take the sum of the moduli of the M–O displacements along the normal mode *q*:
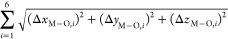
5where (Δ*x*_M–O,*i*_, Δ*y*_M–O,*i*_, Δ*z*_M–O,*i*_)^T^, i.e. the differences of the metal and the *i*-th oxygen displacement coordinates associated with normal mode *q* (with normalized corresponding displacement vectors).

*Ru(acac)_3_*. To carry out the first vibrational spectroscopic investigation of Ru(acac)_3_ at moderate resolution, we synthesized grams of pure Λ and Δ enantiomers (following the recipe detailed in the Supporting Information) and recorded the Fourier transform infrared spectrum of Λ-Ru(acac)_3_ trapped in solid neon at 3 K. Such low-temperature matrix-isolation measurements are not muddled by rotations, which are mostly inhibited, and exhibit narrower bands than the more traditional room-temperature studies in the liquid or solid phase. Importantly, the obtained vibrational band centers are typically shifted by only a few wavenumbers (0.3% of the transition frequency at most; see the Supporting Information) with respect to the gas-phase conditions required for the PV measurements, a level of uncertainty that the current theory cannot provide. The quantum cascade lasers (QCLs) that will be used in the PV measurements typically cover a few wavenumbers. Thus, in combination with theoretical guidance on the optimal vibrational modes for measurements, this precursor spectroscopic characterization is crucial for designing the laser system.

Figure 2 compares the calculated harmonic frequencies with the matrix-isolation measurement. Details on the spectroscopy, and in particular on the assignment of the observed bands to the corresponding internal modes, can be found in the Supporting Information. Overall, the calculated and the measured spectra are in very good agreement.

**Figure 2:**
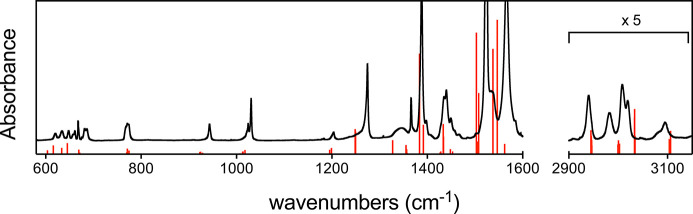
Comparison between the calculated vibrational spectrum (red lines) of Ru(acac)_3_ from the geometry optimization and the infrared Fourier transform spectrum recorded in solid neon at 3 K (above 2800 cm^–1^ intensities and absorbance were multiplied by a factor of 5). The calculated harmonic frequencies were scaled with a scaling factor of 0.969 for comparison with the measured spectrum (see details in the Supporting Information). The baseline of the experimental spectrum is vertically shifted for clarity.

The calculated PV shifts are plotted against the indicator of the M–O displacement (eq 5) in Figure 3 for Ru(acac)_3_. A clear correlation between the magnitude of the displacement and the size of the calculated PV shift can be observed. This demonstrates the potential of this simple yet effective metric to guide the search for viable normal modes. This selection criterion is novel, and it can be extended to other compounds.

**Figure 3:**
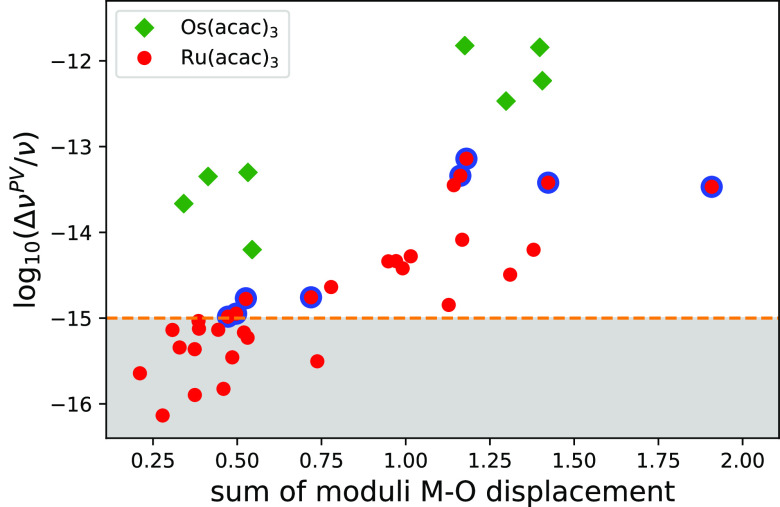
Calculated relative PV frequency shifts (Δν^PV^/ν) of several vibrational transitions in Ru(acac)_3_ and Os(acac)_3_ as a function of the indicator (sum of moduli of Ru–O or Os–O displacements; see text). The red dots represent Ru(acac)_3_, with the larger dots highlighted in blue corresponding to the selected normal modes shown in Table 1; the green diamonds represent the corresponding normal modes of Os(acac)_3_ shown in Table 2. The orange dotted horizontal line is the expected sensitivity attainable by ultrahigh resolution vibrational spectroscopy, bordering the gray zone inaccessible to measurements.

To compare the vibrational frequencies of two enantiomers, we are constructing a Ramsey interferometer that combines a buffer-gas-cooled molecular beam and frequency-stabilized metrology-grade lasers ultimately calibrated to cesium fountain clocks which realize the SI standard of frequency and project a 10^–15^ relative sensitivity on the frequency difference.^25^ In addition to the magnitude of the PV frequency shifts, a number of experimental considerations, thus, have to be taken into account when selecting the vibrational modes for measurements. The intensity of the selected mode should be high enough to make the measurement feasible, and its frequency should lie in a range accessible to current laser technologies. The group at LPL in Paris has demonstrated CO_2_ lasers^51^ and QCLs^25,52−54^ of record frequency stabilities (better than 10^–15^ at 1 s in fractional value) and accuracies (potentially reaching the 2 × 10^–16^ ultimate accuracy of the Cs fountains) necessary for measuring the tiniest PV frequency differences. The CO_2_ lasers, until recently the only available stable sources for precise mid-infrared spectroscopy, span the 9–11 μm range. Mid-infrared QCLs are commercially available in the 4–13 μm range and more sporadically up to 17 μm.

Based on the above criteria (in terms of a large predicted PV shift, intensity, and desired wavelength range), we display a number of promising normal modes in Table 1. All these modes have a relative PV frequency shift Δν^PV^/ν of 10^–15^ or above, which is the sensitivity aimed for in the LPL experiment. For comparison, the PV shift of CHFClBr, a molecule on which much of the experimental work has been conducted so far, has a predicted Δν^PV^/ν ≈ −8 × 10^–17^.^28,29^

**Table 1:** Selected Normal Modes of Ru(acac)_3_ Promising for PV Measurements: Possessing Either Particularly Large Predicted PV Shifts on the 10^–14^ Level (Modes 17–29), or a Large Shift in Combination with a Frequency in the Range of Current Laser Systems (Modes 52 and 53), or Very Large Infrared Intensity (IR int.) (Modes 100 and 102)^a^

normal mode	ν [cm^–1^]	IR int. [km/mol]	Δν^PV^ [mHz]	|Δν^PV^/ν|
17	182	0.009	–449	7.2 × 10^–14^
19	201	1.718	–298	4.6 × 10^–14^
20	223	0.065	279	3.8 × 10^–14^
29	327	7.884	325	3.4 × 10^–14^
52	953	9.564	–30	1.0 × 10^–15^
53	954	1.793	–33	1.1 × 10^–15^
100	1587	453.5	–83	1.7 × 10^–15^
102	1612	44.22	–111	2.3 × 10^–15^

a The calculated harmonic vibrational frequencies (ν) were obtained from the frequency analysis.

Normal modes 52 and 53 are in the laser window currently available at LPL, are reasonably infrared active, and have a predicted PV shift on the 10^–15^ level. The C–O stretch vibrational modes 100 and 102 look even more promising, given their remarkably high intensity, their twice higher predicted relative PV shift, and the commercial availability of QCLs in the corresponding spectral window. Finally, normal modes 17, 19, 20, and 29 should not be overlooked, not only because of their record 10^–14^ relative predicted shift but also because of their lower frequencies, which may prevent the onset of intramolecular vibrational energy redistribution that could obscure the spectra at higher frequencies. However, proper radiation sources are still unavailable in this spectral window.

*Os(acac)_3_*. Our calculated frequencies for Os(acac)_3_ are in good agreement with the vibrational spectrum recorded by Dallmann and Preetz.^46^ Details on this comparison can be found in the Supporting Information. For Os(acac)_3_, calculations were performed for the normal modes that correspond to those of Ru(acac)_3_ displayed in Table 1 (details in the Supporting Information).

The results for Os(acac)_3_ are presented in Table 2 and in Figure 3. For all of the presented normal modes, the signs of the PV shifts are the same for the Ru and Os complexes. This emphasizes the similarity of the vibrational modes between the two complexes and the robustness of the PV shift under slight geometric changes. A striking observation is that the relative PV shifts |Δν^PV^/ν| in Os(acac)_3_ are more than an order of magnitude larger than those in Ru(acac)_3_, reaching 10^–12^ levels, the highest relative PV sensitivities predicted to this day. A closer look at the ratio of absolute shifts reveals, for some of the transitions, a significant enhancement beyond the *Z*^5^ scaling amounting to . This is not entirely unexpected, as the *Z*^5^ dependence was derived for the absolute PV energy shifts^39^ rather than for vibrational transitions; furthermore, similar beyond-the-*Z*^5^ scaling was observed in chiral uranium compounds by Wormit et al.^35^

**Table 2:** PV Shifts of Vibrational Normal Modes in Os(acac)_3_ and a Comparison to Ru(acac)_3_^a^

mode(Os)	ν [cm^–1^]	IR int. [km/mol]	Δν^PV^ [Hz]	|Δν^PV^/ν|	mode(Ru)	ϕ	Δν^PV^(Os)/Δν^PV^(Ru)
16	191	0.091	–9.72	1.5 × 10^–12^	17	0.996	21.7
19	211	2.356	–9.59	1.4 × 10^–12^	19	0.961	32.2
20	224	0.013	4.30	5.9 × 10^–13^	20	0.948	15.4
29	308	2.392	3.09	3.4 × 10^–13^	29	0.838	9.5
52	952	0.464	–1.47	5.0 × 10^–14^	52	0.830	48.4
53	954	1.272	–1.32	4.5 × 10^–14^	53	0.831	40.1
100	1563	245.6	–1.04	2.2 × 10^–14^	100	0.955	12.7
102	1589	102.2	–0.31	6.3 × 10^–15^	102	0.985	3.6

a Normal modes in the two compounds were matched to each other according to their overlap ϕ as defined in eq 1 in the Supporting Information. The harmonic vibrational frequencies (ν) were obtained from the frequency analysis.

Here, it has very favorable consequences for the experiment; the two transitions in Table 2 that lie in the laser window currently available at LPL (modes 52 and 53) experience an enhancement of a factor of 48 and 40, respectively, when changing from Ru(acac)_3_ to Os(acac)_3_, significantly larger than the *Z*^5^ scaling used for a rough estimate would predict. This enhancement pushes the |Δν^PV^/ν| of these modes into the ∼10^–14^ regime, making it much easier to detect at the level of sensitivity already demonstrated at LPL.^23,55^ For such mid-infrared transitions for which proper laser technologies are readily available, this is the highest sensitivity to PV predicted among the various compounds currently at disposal for measurements. This finding provides us with a strong motivation to synthesize this compound and bring it into the gas phase.

In conclusion, we have calculated the PV vibrational frequency shifts for a selection of normal modes in Ru(acac)_3_, a highly promising candidate species for the first detection of parity violation in molecules. We have derived a simple scheme for identifying the most promising vibrational modes that allowed us to pinpoint transitions with exceptionally large PV shifts of hundreds of mHz (corresponding to relative frequency shifts on the order of 10^–15^–10^–14^). These effects are well within the projected sensitivity of the experiment being built at LPL. From the point of view of experimental control, Ru(acac)_3_ benefits from many advantages compared to previously proposed species. It is a robust, readily available archetypal molecule in organic chemistry and the first heavy chiral candidate species for measuring PV that can be brought in the gas phase in a controlled way. This allowed us to report in this work the first spectroscopic investigations of the vibrational spectrum of this molecule at moderate resolution.

Furthermore, the scaling of the vibrational PV shifts with atomic number *Z* was investigated by comparison with Os(acac)_3_, where osmium is the heavier homologue of ruthenium. Here, we find enhancements of the PV shifts that exceed the prediction following from the naive *Z*^5^ scaling, leading to the highest sensitivities to PV among the various compounds that are at our disposal for measurements. This is especially auspicious for the experiment—the investigated modes that fall in the accessible laser windows are enhanced over 40-fold to the 10^–14^ fractional shift regime, making detection feasible within the current experimental sensitivity. Os(acac)_3_ can be synthesized,^46^ and the next experimental steps are to investigate its enantiomeric resolution and its stability upon heating for bringing it into the gas phase for the ultrahigh-resolution spectroscopic studies.

The favorable findings of our theory-experiment investigations pave the way toward first detection of PV effects in the vibrational spectra of chiral molecules realistically measurable at LPL. Identifying the best molecule/transition requires, of course, further significant experimental efforts. The measurement uncertainty will ultimately depend on the resolution and the signal-to-noise ratio obtained for a specific transition. These are affected by a number of experimental considerations, including the line intensity, the number of molecules probed, the rotational and hyperfine structure, and the broadening resulting from intramolecular vibrational energy redistribution, which have yet to be investigated.

Finally, we stress that ^99^Ru, ^101^Ru, ^187^Os, and ^189^Os nuclei are NMR active and, thus, the Ru(acac)_3_ and Os(acac)_3_ complexes potentially open up a possibility for gas-phase NMR measurements.

## Supporting Information Available

Experimental details on the synthesis and neon matrix-isolation Fourier transform infrared spectroscopy of Ru(acac)_3_, full computational details of the PV shifts calculations, comparison of theoretical and experimental vibrational spectra for both Ru(acac)_3_ and Os(acac)_3_, an assignment of the observed vibrational bands for Ru(acac)_3_, and an analysis of the robustness of the calculations.

(PDF)
Transparent Peer Review report available (PDF)
