# Supplementary material for: Toward Detection of the Molecular Parity Violation in Chiral Ru(acac)3 and Os(acac)3
Source: J Phys Chem Lett. 2022 Oct 20;13(42):10011–7. doi: 10.1021/acs.jpclett.2c02434 (PMC9620138; doi:10.1021/acs.jpclett.2c02434)
Supplement: Supplementary file 1 — jz2c02434_si_001.pdf [file jz2c02434_si_001.pdf]

Supporting Information: Toward Detection of the Molecular Parity Violation in Chiral Ru(acac)$_3$ and Os(acac)$_3$

Marit R. Fiechter,[†,‡] Pi A.B. Haase,[†] Nidal Saleh,[¶,§] Pascale Soulard,[∥] Benoît Tremblay,[∥] Remco W.A. Havenith,[⊥,#,@] Rob G.E. Timmermans,[†] Peter Schwerdtfeger,[△] Jeanne Crassous,[§] Benoît Darquié,[∇] Lukáš F. Pašteka,[†,††] and Anastasia Borschevsky[*,†]

†*Van Swinderen Institute for Particle Physics and Gravity (VSI), University of Groningen, 9747 AG Groningen, The Netherlands*

‡*Department of Physics, ETH Zürich, 8093 Zurich, Switzerland*

¶*Department of Organic Chemistry, University of Geneva, 1211 Geneva 4, Switzerland*

§*Université de Rennes, CNRS, ISCR-UMR 6226, Campus de Beaulieu, 35042 Rennes Cedex, France*

∥*Sorbonne Université, CNRS, UMR 8233, MONARIS, F-75005, Paris, France*

⊥*Zernike Institute for Advanced Materials, University of Groningen, 9747 AG, Groningen, The Netherlands*

#*Stratingh Institute for Chemistry, University of Groningen, 9747 AG, Groningen, The Netherlands*

@*Ghent Quantum Chemistry Group, Department of Chemistry, Ghent University, B-9000 Ghent, Belgium*

△*Centre for Theoretical Chemistry and Physics, The New Zealand Institute for Advanced Study, Massey University, 0745 Auckland, New Zealand*

∇*Laboratoire de Physique des Lasers, Université Sorbonne Paris Nord, CNRS, 93430 Villetaneuse, France*

††*Department of Physical and Theoretical Chemistry, Faculty of Natural Sciences, Comenius University, 84215 Bratislava, Slovakia*

E-mail: a.borschevsky@rug.nl

1 Ru(acac)$_3$ synthesis

Large quantities (grams) of pure Δ and Λ enantiomers of Ru(acac)$_3$ were synthesized for this work following an already known procedure,[1] *i.e.* by mixing 2.4 g of rac-Ru(acac)$_3$ (6 mmol, 1 equivalent) with 5.6 g of L-(-)-dibenzoyl-tartaric acid monohydrate (15 mmol, 2.5 equivalents) in 195 mL of a 1:2 benzene/cyclohexane solvent mixture. After stirring for two days, the precipitate was filtered off, redissolved in dichloromethane and treated with aqueous sodium bicarbonate ($NaHCO_3$), to yield Δ-Ru(acac)$_3$ (1.08 g, 45% yield). Similarly, the mother liquors were dried, redissolved in dichloromethane and treated with aqueous sodium bicarbonate, yielding Λ-Ru(acac)$_3$ (1.15 g, 46% yield).

2 Neon matrix-isolation Fourier transform infrared spectroscopy of Ru(acac)$_3$: experimental details

The experimental setup for matrix-isolation Fourier transform infrared spectroscopic investigations has been described in detail previously.[2] The samples are prepared by co-condensing a vapour of Ru(acac)$_3$ together with neon gas onto a highly polished Rh-plated copper mirror maintained at 3 K by a closed-cycle cryogenerator (model PT405, Cryomech) placed inside a stainless steel vacuum chamber (base pressure of $< 5 \times 10^{-7}$ mbar). The vapor of Ru(acac)$_3$ is produced in an oven, at temperatures in the 50 – 200 °C range (the data shown in Figure 2 of the main text have been obtained at an oven temperature of 150 °C for Λ-Ru(acac)$_3$).

The oven is made of an 8 mm diameter 40 mm long stainless steel tube, sealed at both ends, with a 1 mm diameter hole in it. The deposition rate is 6 cm^3/min for high-purity neon (Air Liquide; 99.995 %). A CsI window separates the evacuated interferometer (10^{-3} mbar) from the cryogenic cell (10^{-7} mbar). After typically 10 min of deposition, infrared spectra spanning the 500 – 4000 cm^{-1} window are recorded at 0.1 cm^{-1} resolution using a Bruker 120 FTIR spectrometer and suitable combinations of a Ge/KBr beamsplitter and either an InSb or HgCdTe liquid-nitrogen-cooled detector. Figure 2 of the main text corresponds to a typical average of 100 scans. Bare mirror backgrounds are used as references in processing the sample spectra. The spectra were subsequently subjected to baseline correction to compensate for infrared light scattering and interference patterns.

Low-temperature matrix-isolation measurements are precursor spectroscopic characterization studies leading to the parity violation measurement. In fact, the vibrational band centres obtained are shifted by a few wavenumbers at most with respect to gas-phase measurements, a level of uncertainty that the theory can simply not provide. To illustrate this, we have recorded a matrix-isolation Fourier transform infrared spectrum of trioxane, a species for which a gas phase study has been reported in the literature.[3] Table S1 compares the vibrational band centre frequencies measured in solid neon at 3 K, and in the gas phase. Differences corresponding to at most 0.3% of the transition frequency are observed.

Table S1: Comparison between vibrational band centre frequencies obtained in solid neon at 3 K and in the gas phase for trioxane, and assignment of the observed bands to the corresponding vibrational modes (γ refers to out-of-plane bending, ν to stretching, τ to ring distortion, t to twisting, ω to wagging, and the subscripts a and s to symmetric and antisymmetric vibrations respectively).

**gas phase frequency ($\pm$0.2 cm^{-1})[3]**	**Ne matrix frequency (cm^{-1})**	**gas – matrix frequency (cm^{-1})**	**assignment**
466.2	466.70	–0.5	δCOC, γCH_2, τ
524.2	524.51	–0.3	δOCO, δCOC
944.5	943.05	1.4	γCH_2
977.5	976.80	0.7	ν_sCO, δOCO
1071.8	1070.44	1.4	ν_sCO, ν_aCO
1176.8	1173.64	3.2	ν_sCO, ν_aCO, γCH_2
1305.0	1305.46	–0.5	tCH_2
1409.8	1407.75	2.0	ωCH_2
2851.3	2849.54	1.8	$\nu_s CH_2$
3030.4	3021.27	9.1	$\nu_a CH_2$

3 Computational details

The geometry optimizations were carried out using the Gaussian16 computational chemistry package.[4] As the reference computational scheme, we chose the Def2-TZVPP basis set[5,6] and the B3LYP functional,[7] with Grimme D3 dispersion.[8] Relativistic effects were included by means of the effective-core potential on the central metal atom in the calculation: ECP28MDF on ruthenium[9] and ECP60MDF on osmium.[10]

The relativistic DFT calculations were performed with the DIRAC18 computational program.[11] As the reference for these calculations we take the CAM-B3LYP* functional,[12] which is a Coulomb-attenuated B3LYP functional[13] with parameters optimized for reproducing PV shifts obtained at the coupled cluster level of theory. We used Dyall's v3z basis set on the ruthenium/osmium and oxygen atoms and Dyall's v2z basis set on the carbon and hydrogen atoms.[14,15] In order to reduce the computational effort, these calculations were carried out in the two-component framework, where the Dirac-Coulomb Hamiltonian is transformed into the so-called exact two-component (X2C) Hamiltonian.[16] Two-electron spin-same-orbit interactions are not accounted for in this scheme; these are introduced in a mean-field fashion by the AMFI procedure.[17] The vibrational PV shifts calculated in the X2C procedure have been shown before to differ from those obtained in the full four-component framework by not more than a few percent;[18] this justifies the use of this approximation in our work.

For each chosen mode, eleven single-point relativistic DFT calculations are performed for geometries along the normal mode, yielding the PV energy contribution (as the expectation value of $\hat{H}^{\mathrm{PV}}$) as a function of the normal coordinate, q. We fit a polynomial to these points to obtain the PV potential $V^{\mathrm{PV}}(q)$.

Most of the calculations were performed for the particular set of computational parameters (basis set and DFT functional) listed above which we refer to as the reference. To ensure the robustness and consistency of the results, these parameters were varied for a few normal modes and the results compared. More details can be found in the Section 6 below.

Calculations were performed for analogous normal modes of the two molecules $\mathrm{Ru(acac)_3}$ and $\mathrm{Os(acac)_3}$. In order to identify the corresponding vibrational modes of the two molecules, we take their overlap ϕ, calculated as the normalized dot product of normal coordinates. For the corresponding normal modes, ϕ should approach 1. In this comparison, we found it sufficient to take into account only the displacements of the central metal and the surrounding oxygens (which are the most relevant for the PV shift). The overlap ϕ is normalized by dividing by the geometric mean of the respective displacement vector moduli as:

$$\phi = \frac{\sum_i^{M,\mathrm{O}} \vec{d}_{i,\mathrm{Ru(acac)_3}} \cdot \vec{d}_{i,\mathrm{Os(acac)_3}}}{\sqrt{\sum_i^{\mathrm{Ru,O}} |\vec{d}_{i,\mathrm{Ru(acac)_3}}|^2 \sum_i^{\mathrm{Os,O}} |\vec{d}_{i,\mathrm{Os(acac)_3}}|^2}} \tag{1}$$

Here, $\vec{d}_{i,X}$ denotes the displacement vector of atom i in complex X, and M the metal center of the complex in question.

4 Comparison of the calculated $\mathrm{Ru(acac)_3}$ spectrum with experiment and assignments of the observed vibrational bands

Table S2 compares the calculated (harmonic frequency analysis, B3LYP-D3/Def2-TZVPP, details in Section 3 above) and experimental vibrational frequencies and intensities of the most abundant $\mathrm{Ru(acac)_3}$ isotopologue, and proposes the assignment of the observed bands to internal vibrational modes. The calculated vibrational spectra of the $^{96}\mathrm{Ru}$ and $^{18}\mathrm{O}$ isotopologues have also been used to validate assignments. The experimental intensity corresponds to the integrated absorbance (area under the observed band). It is normalized such that the most intense band at 1565.8 cm^{-1} experimental frequency equals 1000 (a value comparable to the 1032 km/mol calculated intensity). As the frequency analysis was performed in the harmonic approximation, the calculated frequencies are not expected to agree perfectly with the measured frequencies; to correct for this, it is customary to scale the calculated frequencies with a scaling factor which depends on the level of theory and the basis set used (see Refs. 19–23 and references therein). We have thus used the scaling

factor of 0.969 reported by Hanson-Heine[19] for the B3LYP functional in combination with D3(0) dispersion and a 6-311+G(d,p) basis set, which is somewhat smaller but comparable to the Def2-TZVPP basis set used in this work. Generally, the basis set dependence is much weaker compared to the method/functional dependence.[20,21] The same scaling factor of 0.969 was found in other benchmarking studies for calculations performed at the comparable B3LYP/cc-pVTZ[22] and B3LYP/6-311+G(d,p)[23] levels of theory. In all mentioned works, this linear frequency scaling factor was calculated using least squares fitting of the theoretical harmonic frequencies to the experimental frequencies.

5 Comparison of the calculated $Os(acac)_3$ spectrum with experiment

Dallman and Preetz[24] have recorded an infrared Fourier transform spectrum of $Os(acac)_3$ in solid KBr at 10 K. In order to overlay the predicted and the observed spectra, we processed the transmittance infrared spectrum recorded by Dallmann and Preetz; the absorbance spectrum calculated from the measured transmittance is shown in Figure S1, normalized to the maximum peak height. Here again, the calculated harmonic frequencies have been scaled with the scaling factor of 0.969 reported by Hanson-Heine[19] (as discussed in Section 4 above).

Figure S1 shows a reasonable qualitative agreement between our calculations and experiment. Especially the smaller groups of peaks between 1200 cm^{-1} and 600 cm^{-1} seem well predicted. The transitions around 1400 cm^{-1} and 1550 cm^{-1} also show acceptable agreement between theory and measurement. Additionally, since the transmission axis of the experimental spectrum in the publication by Dallmann and Preetz[24] was missing a scale, there may be a slight error in the relative heights of the resulting experimental absorption peaks shown in Figure S1. This however does not influence the peak positions.

Table S2: Comparison between the calculated and experimental vibrational frequencies and intensities of Ru(acac)$_3$, and assignment of the observed bands to the corresponding internal modes (ν refers to stretching, δ to in-plane bending, *tor* to a torsion, τ to an out-of-plane deformation.). The potential energy distribution (PED) is given for values >10%.

**experimental frequency (cm^{-1})**	**experimental intensity**	**calculated frequency (cm^{-1})**	**calculated frequency scaled (cm^{-1})**	**calculated intensity (km/mol)**	**exp − calc frequency (cm^{-1})**	**assignment and PED values (%)**
559.1	2	568<br>569<br>571	551<br>551<br>553	3<br>0<br>0<br>$\Sigma = 3$	8<br>8<br>6	τCCOC (68), *tor*HCCC (11)
620.3	24	623	604	17	16	δCCO (39), νRuO (15), δCORu (10)
634.2	36	636	616	38	18	δCCO (31)
648.1	24	654	634	27	14	δCORu (31)
657.5	12	666	645	48	12	δCCO (13)
661.3	9	680	659	0	2	τCCOC (34), *tor*CCC (15)
668.4	17	684	663	3	5	
681.4	31	691	669	19	12	δCCO (19), νCC (15), δCCC (12)
686.5	27	693<br>698	671<br>676	7<br>1<br>$\Sigma = 8$	15<br>10	τCCOC (27)<br>δCCO (23)
768.5	15	795	770	9	–2	*tor*HCCC (86)
771.3	31	796	771	24	0	
774.6	30	799	775	16	0	
943.5	42	953<br>954<br>954<br>957<br>958<br>959	923<br>924<br>925<br>927<br>929<br>929	10<br>2<br>11<br>2<br>6<br>1<br>$\Sigma = 32$	20<br>19<br>18<br>16<br>14<br>14	νCC (66)<br>νCC (36), *tor*HCCC (14), δCCO (10)
1024.4	26	1040<br>1045 (×2)<br>1046 (×3)	1007<br>1012<br>1013 (×2)<br>1014 (×2)	4<br>0<br>10, 11<br>7, 12<br>$\Sigma = 44$	17<br>12<br>11 (×2)<br>10 (×3)	*tor*HCCC (42), δHCH (15)
1030.7	63	1050 (×2)<br>1052	1018 (×2)<br>1019	16, 18<br>0<br>$\Sigma = 34$	13 (×2)<br>12	
unobserved	0	1059 (×3)	1026 (×3)	0, 1, 0		*tor*HCCC (55), δHCH (13), τCCOC (12)
1203.8	17	1234<br>1236<br>1237	1195<br>1197<br>1199	19<br>5<br>27<br>$\Sigma = 51$	9<br>7<br>5	δHCC (53), νCC (21)
1274.5	146	1289<br>1290<br>1294	1249<br>1250<br>1254	108<br>98<br>1<br>$\Sigma = 207$	25<br>24<br>20	νCC (56)
1345.0	56	1370	1327	60	18	νOC (69), *tor*HCCC (10)
1366.1	34	1399 (×2)<br>1400<br>1401 (×2)<br>1402	1355<br>1356<br>1357 (×2)<br>1358 (×2)	39<br>12<br>21, 1<br>5, 2<br>$\Sigma = 80$	11<br>10<br>9 (×2)<br>8 (×2)	δHCH (83)
1388.1	407	1428<br>1436	1384<br>1392	433<br>127<br>$\Sigma = 560$	4<br>–4	νCC (63)<br>νCC (54), δHCH (16)
1428.0	25	1473 (×3)	1427 (×2)<br>1428	7, 8<br>0<br>$\Sigma = 15$	1 (×2)<br>0	δHCH (68), *tor*HCCC (17)
1435.3	69	1474 (×3)	1428 (×3)	11, 9, 12<br>$\Sigma = 32$	7 (×3)	δHCH (71)
1440.0<br>1449.6	182<br>67<br>$\Sigma = 249$	1479<br>1480<br>1481	1433<br>1434<br>1435	130<br>75<br>0<br>$\Sigma = 205$	7 / 17<br>6 / 16<br>5 / 15	δHCH (62), *tor*HCCC (14)
1458.0<br>1466.0	14<br>14<br>$\Sigma = 28$	1489<br>1495<br>1500	1443<br>1448<br>1453	0<br>22<br>12<br>$\Sigma = 34$	15 / 23<br>10 / 18<br>5 / 13	δHCH (57), *tor*HCCC (17)
1522.5<br>1525.0	484<br>223<br>$\Sigma = 707$	1551<br>1553<br>1556	1503<br>1504<br>1508	523<br>55<br>263<br>$\Sigma = 841$	19 / 22<br>18 / 21<br>14 / 17	νCC (39), δHCC (35)
1565.8	1000	1587<br>1596	1538<br>1547	454<br>578<br>$\Sigma = 1032$	28<br>19	νOC (63)<br>νCC (63)
1584.3	26	1612	1562	44	4	νOC (56)
2940.2	45	3039 (×2)<br>3040 (×2)<br>3041 (×2)	2945 (×3)<br>2946 (×2)<br>2947	21, 1, 16<br>9, 12<br>8<br>$\Sigma = 66$	–5 (×3)<br>–6 (×2)<br>–7	νCH (91)
2982.5	34	3096 (×2)<br>3097 (×2)<br>3099 (×2)	3000 (×2)<br>3001 (×2)<br>3003 (×2)	3, 8<br>0, 12<br>3, 9<br>$\Sigma = 35$	–17 (×2)<br>–18 (×2)<br>–20 (×2)	νCH (97)
3008.6<br>3019.6	64<br>22<br>$\Sigma = 86$	3130 (×3)<br>3131 (×3)	3033 (×3)<br>3034 (×3)	31, 1, 39<br>4, 16, 1<br>$\Sigma = 92$	–24 / –13 (×3)<br>–25 / –14 (×3)	νCH (88)
3081.0<br>3095.4	6<br>22<br>$\Sigma = 28$	3203<br>3206 (×2)	3104<br>3106 (×2)	13<br>20, 3<br>$\Sigma = 36$	–23 / –9<br>–25 / –11	νCH (99)

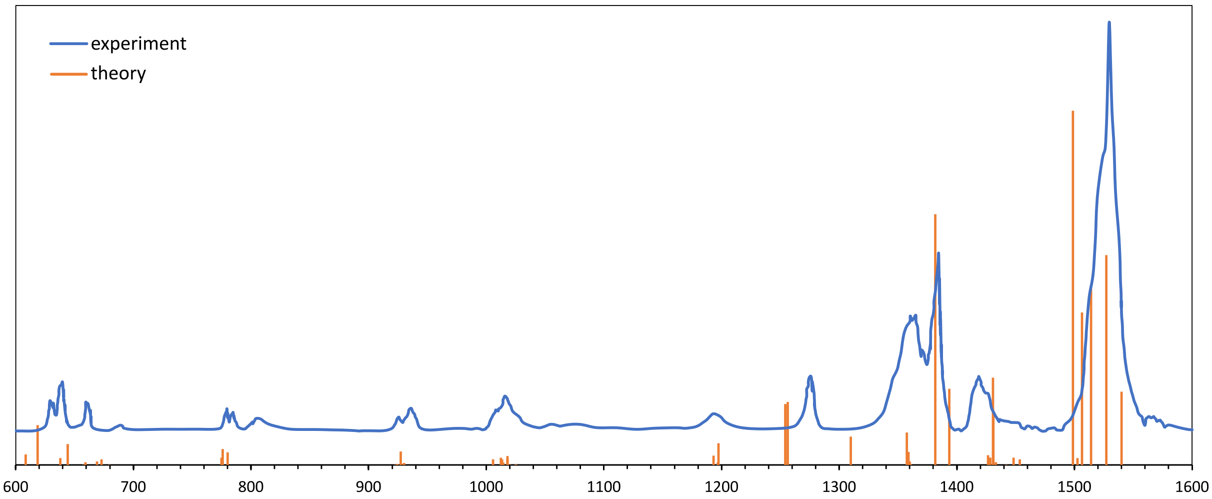

Figure S1: Comparison between experimental and calculated vibrational spectra of $Os(acac)_3$. The theoretical spectrum is horizontally scaled by a factor 0.969. The baseline of the experimental spectrum is vertically shifted for clarity.

6 Robustness of the calculations

The computational parameters were individually varied for six different normal modes in $Ru(acac)_3$ to investigate the robustness and the consistency of the obtained results. We selected these 6 modes based on their large calculated PV shifts from lower and higher frequency range. We have investigated three effects in the the geometry optimization step – basis set (Def2-SVP[5] instead of Def2-TZVPP), different DFT functionals (CAM-B3LYP[13] and PBE0[25,26] instead of B3LYP) and exclusion of the Grimme D3 dispersion (which was included in the reference calculation). Similarly, for the relativistic calculations of the PV potential, the basis sets on the metal center and the oxygen atoms were varied (v2z and v4z[14,15] instead of v3z), and different functionals were compared (B3LYP and PBE0 rather than CAM-B3LYP*).

The results of this analysis are shown in Figure S2 as relative differences with respect to the reference method. In case of geometry optimizations, the effect of using the smaller Def2-SVP basis set introduces changes of at most $\sim 25\%$ with the mean absolute deviation (MAD) of 12%. It can be safely assumed that progressing to a larger basis set (i.e. Def2-QZVPP) would introduce much smaller errors and thus the Def2-TZVPP should be entirely sufficient for this study. Much larger effect comes from varying the DFT functional, CAM-B3LYP and PBE0 give MADs of 17% and 26%, respectively, with the individual differences reaching as far as $\sim 70\%$. Importantly, the ordering of the normal modes from large to small PV shifts is largely preserved across different functionals. Excluding the empirical D3

dispersion correction in the geometry optimization has a smaller but not negligible effect with a MAD of 18%.

In case of the relativistic PV energy calculations, we observe a similar behaviour – the basis set effect is smaller compared to the effect of the functional. In fact, the good agreement of the v4z results with the reference values confirms the convergence of the calculated parameters at the v3z level. Swapping the reference CAM-B3LYP* functional for B3LYP and PBE0 gives MADs of 23% and 19%, respectively, again presenting as the dominant source of uncertainty.

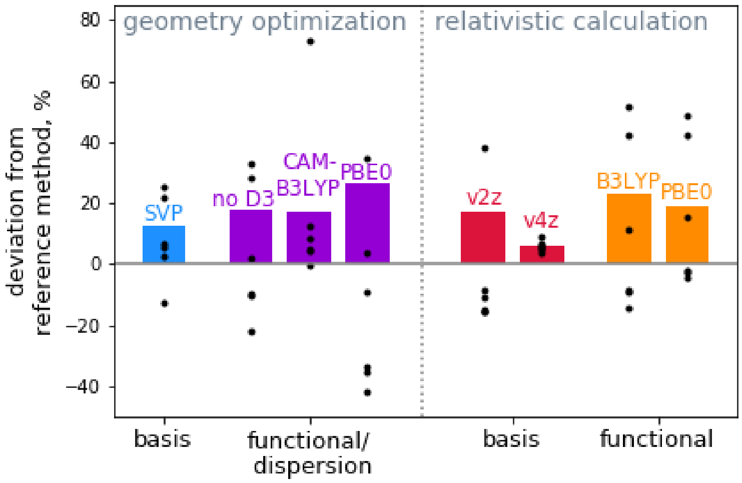

Figure S2: Dependence of the calculated PV shifts in Ru(acac)$_3$ upon the method used relative to the reference methodology – B3LYP-D3/Def2-TZVPP for the geometry optimizations and CAM-B3LYP*/dyall-v3z(v2z) for the relativistic PV calculations. The dots represent the individual normal modes used in the analysis, the bars represent the mean absolute deviations (MAD).

Therefore, overall, we expect the accuracy of our results to be better than an order of magnitude, which is sufficient for providing an indication of feasibility for the planned experiment. This thorough analysis was performed for Ru(acac)$_3$; due to the similar properties of the two molecules, similar robustness can be expected for Os(acac)$_3$.

The data summarized in Figure S2 are detailed below, subdivided into four tables, showing the variation of the calculated PV shift when varying 1) the basis set, and 2) the functional used and the inclusion or exclusion of the Grimme D3 dispersion in the geometry optimization and frequency analysis, and 3) the basis and 4) the functional used for the relativistic calculations. Also, the frequencies resulting from the harmonic frequency analysis in Gaussian (ν_{harmonic}) are shown.

Table S3: Effect of changing the basis set used in the geometry optimization and frequency analysis (calculated using the B3LYP+D3 functional) on the resulting PV shift. The reference calculation is printed in bold.

mode	basis set	$\Delta\nu_{PV}$ (mHz)	Δ from ref.	ν_{harmonic}
19	**Def2-TZVPP**	**–297.9**		200.8
	Def2-SVP	–260.1	–13%	201.2
29	**Def2-TZVPP**	**325.3**		327.2
	Def2-SVP	342.6	5%	326.4
41	**Def2-TZVPP**	**159.1**		635.5
	Def2-SVP	163.5	3%	633.8
100	**Def2-TZVPP**	**–81.5**		1586.7
	Def2-SVP	–99.3	22%	1613.5
101	**Def2-TZVPP**	**–29.0**		1596.2
	Def2-SVP	–36.3	25%	1623.7
102	**Def2-TZVPP**	**–86.3**		1612.1
	Def2-SVP	–91.9	7%	1640.1

Table S4: Effect of changing the functional and dispersion correction used in the geometry optimization and frequency analysis (calculated using the Def2-TZVPP basis set) on the resulting PV shift. The reference calculation is printed in bold.

mode	functional, dispersion	$\Delta\nu_{PV}$ (mHz)	Δ from ref.	ν_{harmonic}
19	**B3LYP+D3**	**–297.9**		200.8
	B3LYP	–232.2	–22%	198.3
	CAM-B3LYP+D3	–322.5	8%	199.3
	PBE0+D3	–400.6	35%	203.1
29	**B3LYP+D3**	325.3		327.2
	B3LYP	294.0	–10%	324.5
	CAM-B3LYP+D3	338.6	4%	334.2
	PBE0+D3	337.4	4%	334.2
41	**B3LYP+D3**	**159.1**		635.5
	B3LYP	142.4	–10%	628.4
	CAM-B3LYP+D3	179.4	13%	647.5
	PBE0+D3	92.6	–42%	642.8
100	**B3LYP+D3**	**–81.5**		1586.7
	B3LYP	–83.0	2%	1586.0
	CAM-B3LYP+D3	–81.3	0%	1639.4
	PBE0+D3	–74.1	–9%	1625.3
101	**B3LYP+D3**	**–29.0**		1596.2
	B3LYP	–38.5	33%	1596.0
	CAM-B3LYP+D3	–50.1	73%	1646.1
	PBE0+D3	–19.2	–34%	1632.6
102	**B3LYP+D3**	**–86.3**		1612.1
	B3LYP	–110.9	29%	1612.3
	CAM-B3LYP+D3	–90.4	5%	1667.6
	PBE0+D3	–55.7	–35%	1651.2

Table S5: Effect of changing the basis set for Ru and O used in the relativistic calculations on the resulting PV shift (calculated using the CAM-B3LYP* functional); the basis set for C and H is kept at v2z for all calculations. The reference calculation is printed in bold.

mode	basis set	$\Delta\nu_{PV}$ (mHz)	Δ from ref.	ν_{harmonic}
19	v2z	–253.0	–15%	200.8
	**v3z**	**–297.9**		200.8
	v4z	–312.4	5%	200.8
29	v2z	274.3	–16%	327.2
	**v3z**	**325.3**		327.2
	v4z	346.4	6%	327.2
41	v2z	134.9	–15%	635.5
	**v3z**	**159.1**		635.5
	v4z	173.8	9%	635.5
100	v2z	–72.7	–11%	1586.7
	**v3z**	**–81.5**		1586.7
	v4z	–86.4	6%	1586.7
101	v2z	–40.1	38%	1596.2
	**v3z**	**–29.0**		1596.2
	v4z	–30.0	4%	1596.2
102	v2z	–79.0	–8%	1612.1
	**v3z**	**–86.3**		1612.1
	v4z	–91.0	6%	1612.1

Table S6: Effect of changing the functional used in the relativistic calculations on the resulting PV shift (calculated using the dyall.v3z/v2z basis sets for Ru, O/C, H). The reference calculation is printed in bold.

mode	functional	$\Delta\nu_{PV}$ (mHz)	Δ from ref.	ν_{harmonic}
19	**CAM-B3LYP***	**–297.9**		200.8
	B3LYP	–451.9	52%	200.8
	PBE0	–423.9	42%	200.8
29	**CAM-B3LYP***	**325.3**		327.2
	B3LYP	298.5	–8%	327.2
	PBE0	311.0	–4%	327.2
41	**CAM-B3LYP***	**159.1**		635.5
	B3LYP	144.8	–9%	635.5
	PBE0	155.4	–2%	635.5
100	**CAM-B3LYP***	**–81.5**		1586.7
	B3LYP	–90.9	12%	1586.7
	PBE0	–94.1	15%	1586.7
101	**CAM-B3LYP***	**–29.0**		1596.2
	B3LYP	–41.3	42%	1596.2
	PBE0	–43.1	49%	1596.2
102	**CAM-B3LYP***	**–86.3**		1612.1
	B3LYP	–73.8	–15%	1612.1
	PBE0	–84.5	–2%	1612.1

Bibliography

(1) Drake, A.; Gould, J. M.; Mason, S.; Rosini, C.; Woodley, F. The optical resolution of tris(pentane-2,4-dionato)metal(III) complexes. *Polyhedron* **1983**, *2*, 537–538.

(2) Danset, D.; Manceron, L. Mid- and near-IR electronic absorption spectrum of CoO isolated in solid neon. Vibronic data for low-lying electronic states. *J. Phys. Chem. A* **2003**, *107*, 11324–11330.

(3) Churakov, V.; Fuss, W. Isotopically selective IR multiphoton dissociation of 1,3,5-trioxane. *Appl. Phys. B* **1996**, *62*, 203–212.

(4) Frisch, M. J. et al. Gaussian 16 Revision B.01. 2016; Gaussian Inc. Wallingford CT.

(5) Weigend, F.; Ahlrichs, R. Balanced basis sets of split valence, triple zeta valence and quadruple zeta valence quality for H to Rn: Design and assessment of accuracy. *Phys. Chem. Chem. Phys.* **2005**, *7*, 3297–3305.

(6) Weigend, F. Accurate Coulomb-fitting basis sets for H to Rn. *Phys. Chem. Chem. Phys.* **2006**, *8*, 1057–1065.

(7) Stephens, P.; Devlin, F.; Chabalowski, C.; Frisch, M. *Ab initio* calculation of vibrational absorption and circular dichroism spectra using density functional force fields. *J. Phys. Chem.* **1994**, *98*, 11623–11627.

(8) Grimme, S.; Antony, J.; Ehrlich, S.; Krieg, H. A consistent and accurate *ab initio* parametrization of density functional dispersion correction (DFT-D) for the 94 elements H-Pu. *J. Chem. Phys.* **2010**, *132*, 154104.

(9) Peterson, K.; Figgen, D.; Dolg, M.; Stoll, H. Energy-consistent relativistic pseudopotentials and correlation consistent basis sets for the 4d elements Y–Pd. *J. Chem. Phys.* **2007**, *126*, 124101.

(10) Figgen, D.; Peterson, K. A.; Dolg, M.; Stoll, H. Energy-consistent pseudopotentials and correlation consistent basis sets for the 5d elements Hf–Pt. *J. Chem. Phys.* **2009**, *130*, 164108.

(11) DIRAC, a relativistic *ab initio* electronic structure program, Release DIRAC18 (2018), written by T. Saue, L. Visscher, H. J. Aa. Jensen, and R. Bast, with contributions from V. Bakken, K. G. Dyall, S. Dubillard, U. Ekström, E. Eliav, T. Enevoldsen, E. Faßhauer, T. Fleig, O. Fossgaard, A. S. P. Gomes, E. D. Hedegård, T. Helgaker, J. Henriksson, M. Iliaš, Ch. R. Jacob, S. Knecht, S. Komorovský, O. Kullie, J. K. Lærdahl, C. V. Larsen, Y. S. Lee, H. S. Nataraj, M. K. Nayak, P. Norman, G. Olejniczak, J. Olsen, J. M. H. Olsen, Y. C. Park, J. K. Pedersen, M. Pernpointner, R. di Remigio, K. Ruud, P. Sałek, B. Schimmelpfennig, A. Shee, J. Sikkema, A. J. Thorvaldsen, J. Thyssen, J. van Stralen, S. Villaume, O. Visser, T. Winther, and S. Yamamoto (available at `https://doi.org/10.5281/zenodo.2253986`, see also `http://www.diracprogram.org`).

(12) Thierfelder, C.; Rauhut, G.; Schwerdtfeger, P. Relativistic coupled-cluster study of the parity-violation energy shift of CHFClBr. *Phys. Rev. A* **2010**, *81*, 032513.

(13) Yanai, T.; Tew, D.; Handy, N. A new hybrid exchange–correlation functional using the Coulomb-attenuating method (CAM-B3LYP). *Chem. Phys. Lett.* **2004**, *393*, 51–57.

(14) Dyall, K. Relativistic double-zeta, triple-zeta, and quadruple-zeta basis sets for the light elements H–Ar. *Theor. Chem. Acc.* **2016**, *135*, 128.

(15) Dyall, K. Relativistic double-zeta, triple-zeta, and quadruple-zeta basis sets for the 5d elements Hf–Hg. *Theor. Chem. Acc.* **2004**, *112*, 403–409, revision K.G. Dyall, K.G; Gomes, A.S.P., *Theor. Chem. Acc.* **2009**, *125*, 97–100.

(16) Iliaš, M.; Saue, T. An infinite-order two-component relativistic Hamiltonian by a simple one-step transformation. *J. Chem. Phys.* **2007**, *126*, 064102.

(17) Schimmelpfennig, B. AMFI, an atomic mean-field spin-orbit integral program. *Stockholm University* **1996**,

(18) Figgen, D.; Koers, A.; Schwerdtfeger, P. NWHClI: a small and compact chiral molecule with large parity-violation effects in the vibrational spectrum. *Angew. Chem. Int. Edit.* **2010**, *49*, 2941–2943.

(19) Hanson-Heine, M. Benchmarking DFT-D dispersion corrections for anharmonic vibrational frequencies and harmonic scaling factors. *J. Phys. Chem. A* **2019**, *123*, 9800–9808.

(20) Irikura, K. K.; Johnson, R. D.; Kacker, R. N.; Kessel, R. Uncertainties in scaling factors for ab initio vibrational zero-point energies. *J. Chem. Phys.* **2009**, *130*, 114102.

(21) Scott, A. P.; Radom, L. Harmonic Vibrational Frequencies: An Evaluation of Hartree-Fock, Møller-Plesset, Quadratic Configuration Interaction, Density Functional Theory, and Semiempirical Scale Factors. *J Phys. Chem.* **1996**, *100*, 16502–16513.

(22) Sinha, P.; Boesch, S. E.; Gu, C.; Wheeler, R. A.; Wilson, A. K. Harmonic Vibrational Frequencies: Scaling Factors for HF, B3LYP, and MP2 Methods in Combination with Correlation Consistent Basis Sets. *J Phys. Chem. A* **2004**, *108*, 9213–9217.

(23) Merrick, J. P.; Moran, D.; Radom, L. An Evaluation of Harmonic Vibrational Frequency Scale Factors. *J Phys. Chem. A* **2007**, *111*, 11683–11700.

(24) Dallmann, K.; Preetz, W. Darstellung, Kristallstruktur, Schwingungsspektren und Normalkoordinatenanalyse von Os(acac)$_3$/Synthesis, crystal structure, vibrational spectra, and normal coordinate analysis of Os(acac)$_3$. *Z. Naturforsch. B* **1998**, *53*, 232–238.

(25) Perdew, J.; Burke, K.; Ernzerhof, M. Generalized gradient approximation made simple. *Phys. Rev. Lett.* **1996**, *77*, 3865–3868.

(26) Adamo, C.; Barone, V. Toward reliable density functional methods without adjustable parameters: The PBE0 model. *J. Chem. Phys.* **1999**, *110*, 6158–6170.
