# Supplementary material for: Toward Detection of the Molecular Parity Violation in Chiral Ru(acac)3 and Os(acac)3
Source: J Phys Chem Lett. 2022 Oct 20;13(42):10011–7. doi: 10.1021/acs.jpclett.2c02434 (PMC9620138; doi:10.1021/acs.jpclett.2c02434)
Supplement: Supplementary file 2 — jz2c02434_si_002.pdf [file jz2c02434_si_002.pdf]

jz-2022-02434d.R1

Name: Peer Review Information for "Towards Detection of the Molecular Parity Violation in Chiral $Ru(acac)_3$ and $Os(acac)_3$"

First Round of Reviewer Comments

Reviewer: 1

Comments to the Author

This is an important and interesting paper presenting accurate calculation of parity violation effect in chiral molecules and preliminary measurements of the vibrational spectrum. This is necessary to select transitions sensitive to the parity violation effect. The authors concluded that the sensitivity of future PV effect measurement will exceed expected magnitude of the effect by two orders of magnitude. This will allow for the first time to measure PV effect in chiral molecules. Previous attempts of such measurements were carried out for many years but did not reach necessary sensitivity.

Reviewer: 2

Comments to the Author

The authors studied the prospects of detecting molecular parity violation in vibrational spectra of chiral Ru(acac)3 and Os(acac)3 molecules. The main part of the work was focused on calculating the sensitivity of vibrational transitions to parity violation. The authors optimized the geometries at the DFT level and next calculated the parity violation potentials along the normal modes using higher-level relativistic DFT methods. Finally, the shifts of a vibrational level were obtained as expectation values of parity violation potentials with vibrational wavefunctions. In the supplementary materials, the authors carefully addressed the accuracy of their calculations. On the experimental side, they presented the infrared Fourier transform spectrum recorded in solid neon at 3 K.

While this is a very nice report of progress toward detecting molecular parity violation in vibrational spectra, I am not convinced if the manuscript is suitable for the highly-selective Journal of Physical Chemistry Letters. The experimental results on their own are rather standard. The theoretical calculations predict and confirm that chiral Ru(acac)3 and Os(acac)3 molecules are good candidates for detecting molecular parity violation in vibrational spectra, but the predicted relative sensitivity is not higher than in other known systems. Finally, the experimental authors already suggested the use of Ru(acac)3 molecules for detecting molecular parity violation in their PCCP 23, 24140 (2021) paper without any predictions on their sensitivity. The manuscript is well written. The methodology and results seem solid.

Therefore, I recommend publication in the Journal of Physical Chemistry A as it is.

My only technical comment is about scaling vibrational frequencies that the authors applied justified as a standard approach. The agreement without scaling would probably still be satisfactory, avoiding unnecessary ambiguity, because the finding of scaling factors was not described in detail enough to reproduce it.

Author's Response to Peer Review Comments:

In our revised manuscript, we have made of all the requested technical editorial changes. We thank both reviewers for their valuable comments on our manuscript. Since Reviewer 1 recommended our paper for publication in JPCL "as is", stating that our research "represents a significant new contribution", in the following, we will focus on concerns raised by Reviewer 2, specifically:

<u>Comment:</u> "the experimental authors already suggested the use of Ru(acac)3 molecules for detecting molecular parity violation in their PCCP 23, 24140 (2021) paper without any predictions on their sensitivity"

<u>Response:</u>
$Ru(acac)_3$ was indeed proposed as a candidate for PV measurements in an earlier publication of some of the present authors. However, the basis for that proposal was its experimental advantages; at that point nothing was known concerning its PV sensitivity save a rough estimate (based on Ru being a relatively heavy element).

The current MS contains several significant advances and points of novelty:

- We perform reliable calculations of the sensitivity of $Ru(acac)_3$ to PV effects. Furthermore, we determine the best vibrational transitions for measurements. We see that the sensitivity to PV can vary by over an order of magnitude between the different transitions in the same molecule and hence one must go beyond estimates in planning the measurements. In the past, we have seen that the wrong choice of transition led to lower significance of the measurements (Gaul et al, PRL 125, 123004)
- We propose a simple scheme that allows us to identify the most promising transitions prior to performing the costly ab initio calculations based on the correlation of fractional PV frequency shifts with the total metal-oxygen displacements. This is novel and can certainly be extended to other systems.
- We go beyond the previously suggested $Ru(acac)_3$ and provide a solid proposal for measurements on its heavier homologue, $Os(acac)_3$, where the sensitivity is even higher.

Based on the Reviewer 2's comments, we make the following changes to improve our text:

- In the bottom line of p. 7, the sentence: "~~*This correlation will guide the search for viable normal modes in similar compounds.*~~" was replaced by: "*It demonstrates the potential of this simple yet effective metric to guide the search for viable normal modes. This selection criterion is novel and it can be extended to other compounds.*"

Comment: "The experimental results on their own are rather standard."

Response:
As stressed in our text, precise spectroscopic data on such heavy chiral compounds (and in particular low-temperature matrix-isolation measurements allowing vibrational frequencies close to gas phase measurements to be obtained) are scarce or simply non-existent. Our work is most probably the first ever such measurement on a highly promising species for the detection of PV. Importantly, our matrix-isolation measurements are carried out with enantiopure samples. This demonstrates an unprecedented level of experimental control on a heavy organo-metallic chiral candidate species for measuring PV (in particular the ability to bring into the gas-phase the proposed systems which start out as solids). These original experimental results make our report the first realistic proposal of a heavy chiral compound for measuring PV.

Comment: "the predicted relative sensitivity is not higher than in other known systems"

Response:
We would like to offer the following counterarguments:

- The sensitivity to PV in the proposed systems is actually the highest predicted so far with far-infrared transitions exhibiting PV frequency shifts at a record 1.5×10^{-12} level (see Table 2).
- For mid-infrared transitions for which proper laser technologies are readily available, the sensitivity to PV is also the highest among the various compounds that are currently at our disposal for measurements. Thus, again, this is the first proposal of a heavy chiral compound that can be realistically measured.

Based on the Reviewer 2's comment we make the following changes to improve our text:

- In the last paragraph of p. 9 – a sentence was modified (addition in red): "*A striking observation is that the relative PV shifts $|\Delta\nu PV/\nu|$ in $Os(acac)_3$ are more than an order of magnitude larger than those in $Ru(acac)_3$, reaching 10^{-12} levels, the highest relative PV sensitivities predicted to this day.*"
- In the 2nd paragraph of p. 10 – a sentence was added: "*For such mid-infrared transitions for which proper laser technologies are readily available, this is the highest sensitivity to PV predicted among the various compounds currently at disposal for measurements.*"
- In the second paragraph of p. 11 – a sentence was modified (addition in red): "*Here, we find enhancements of the PV shifts that exceed the prediction following from the naive Z^5 scaling, leading to the highest sensitivities to PV among the various compounds that are at our disposal for measurements.*"
- In the 3rd paragraph of p. 11 – a sentence was modified (addition in red): "*The favourable findings of our theory-experiment investigations pave the way towards first detection of PV effects in vibrational spectra of chiral molecules realistically measurable at LPL.*"

Comment: "My only technical comment is about scaling vibrational frequencies that the authors applied justified as a standard approach. The agreement without scaling would probably still be satisfactory, avoiding unnecessary ambiguity, because the finding of scaling factors was not described in detail enough to reproduce it."

Response:
Without the scaling, the visual agreement between measured and calculated spectra is not satisfactory. This is illustrated in the following figure displaying the comparison between the calculated vibrational spectrum (red lines) of Ru(acac)$_3$ from the geometry optimization without scaling and the infrared Fourier transform spectrum recorded in solid neon at 3 K (above 2800 cm^{-1} intensities and absorbance were multiplied by a factor of 5):

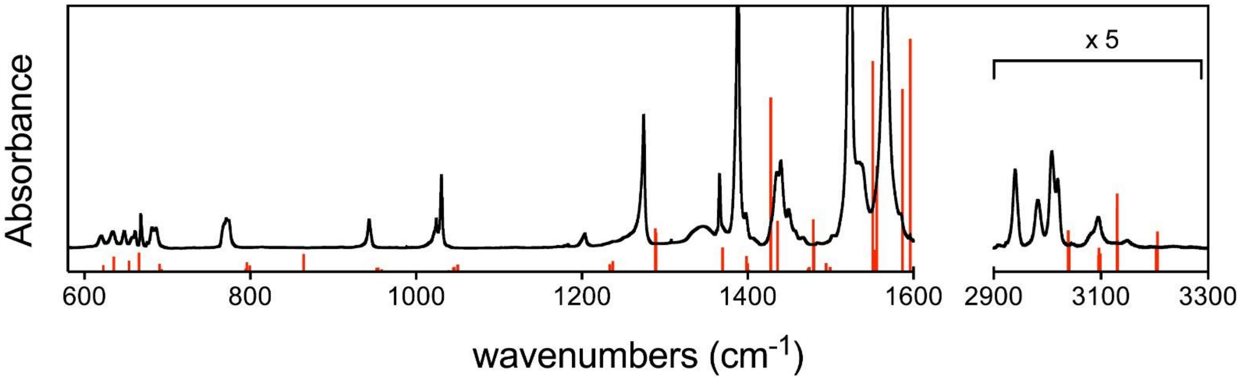

For frequencies in the 2900 – 3300 cm^{-1} range, the theory-experiment agreement is poor and even for frequencies smaller in the 600 – 1700 cm^{-1} range, the agreement is modest. Therefore, we deem the common practice of scaling harmonic frequencies necessary in this work for the benefit of the reader. Nevertheless, we agree with the Reviewer 2 that the finding of scaling factors was not described in detail enough to reproduce it. For simplicity, we have decided to refer more to the literature in order to illustrate that the scaling approach is customary, to source the relevant scaling factor reported in the literature, and to give insights of how it was determined.

Based on the reviewer's comments we make the following changes to improve our text:

- We change Figure 2 of the main text and Figure S1 of the Supporting Information so that the theoretical spectrum in both figures is horizontally scaled by a factor 0.969 and modify the captions of both figures accordingly.
- We update the scaled calculated frequencies in Table S2 of the Supporting Information using the same scaling factor of 0.969.
- We modify the text in Sections 4 and 5 of the Supporting Information adding appropriate references and details on the selection of the scaling factor.

Thus, this MS brings together all the needed ingredients and paves the way to first detection of PV in chiral molecules. We believe that its quality justifies publication in JPC Letters.
